# Loss-of-function variant in *MAGT1* leading to XMEN disease in a Colombian patient with a common variable immunodeficiency

**DOI:** 10.7705/biomedica.7636

**Published:** 2024-12-23

**Authors:** Sebastián Gutiérrez-Hincapié, Julio César Orrego, José Luis Franco, Claudia M. Trujillo-Vargas

**Affiliations:** 1 Grupo de Inmunodeficiencias Primarias, Facultad de Medicina, Universidad de Antioquia, Medellín, Colombia Universidad de Antioquia Grupo de Inmunodeficiencias Primarias Facultad de Medicina Universidad de Antioquia Medellín Colombia

**Keywords:** Common variable immunodeficiency, herpesvirus type 4, human, primary immunodeficiency diseases, inmunodeficiencia común variable, herpesvirus humano de tipo 4, enfermedades de inmunodeficiencia primaria.

## Abstract

**Introduction.:**

Common variable immunodeficiency is a diagnosis of exclusion in immunodeficient patients with increased susceptibility to infections, hypogammaglobulinemia, deficient response to vaccination, or low percentages of switched memory B cells. In low- and middle-income countries, the elucidation and study of molecular defects in these patients may take decades.

**Objective.:**

To elucidate the genetic defect conferring impaired immunity in a patient diagnosed with common variable immunodeficiency.

**Materials and methods.:**

The clinical phenotype was extracted from the clinical records. NKG2D expression in natural killer cells was evaluated by flow cytometry. The whole exome sequencing was performed in the patient and his parents. Sanger sequencing confirmed the pathogenic variant.

**Results.:**

The patient suffered from upper respiratory and urinary tract infections, autoimmune hemolytic anemia, and hepatopathy. NKG2D was decreased in the different blood subpopulations of natural killer cells. Serologic and viral load studies for Epstein- Barr virus were positive, but no B-cell malignancies have been documented. The patient presented a nonsense variant in the exon 3 of the *MAGT1* gen (c.409C>T, rs387906724) in the X chromosome, resulting in an amino acid substitution of arginine for a stop codon in the position 137 of the protein (R137X). The mother also carried the pathogenic variant in a heterozygous state.

**Conclusions.:**

We report the clinical case of the first Colombian male patient with a pathogenic variant in *MAGT1* associated with XMEN disease. Genetic counseling and followup are recommended for families with similar cases to allow prompt detection of new cases.

## Resumen

**Introducción.:**

La inmunodeficiencia común variable es un diagnóstico de exclusión en pacientes con mayor susceptibilidad a las infecciones, hipogammaglobulinemia, reacción deficiente a la vacunación o disminución de los porcentajes de linfocitos B de memoria en sangre. En los países de bajos y medianos ingresos, la definición y el estudio de los defectos moleculares en estos pacientes puede tardar décadas.

**Objetivo.:**

Dilucidar el defecto genético que produce la alteración de la inmunidad en un paciente con diagnóstico de inmunodeficiencia común variable.

**Materiales y métodos.:**

El fenotipo del paciente se obtuvo de la historia clínica. La expresión de NKG2D en células asesinas naturales se evaluó mediante citometría de flujo. Se realizó la secuenciación completa del exoma del paciente y de sus padres. Por medio de secuenciación por Sanger, se confirmó la variante patogénica.

**Resultados.:**

El paciente reportó infecciones urinarias y de las vías aéreas superiores, además de anemia hemolítica autoinmunitaria y hepatopatía. Se observó una disminución de la expresión de NKG2D en las diferentes subpoblaciones sanguíneas de las células asesinas naturales. Los estudios serológicos y de carga viral para el virus de Epstein-Barr fueron positivos, pero, hasta la fecha, no se ha documentado neoplasia de las células B. El paciente presentó una mutación sin sentido en el exón 3 del gen *MAGT1* (c.409C>T, rs387906724) en el cromosoma X, lo que resultó en una sustitución del aminoácido arginina por un codón de parada en la posición 137 de la proteína (R137X).

**Conclusiones.:**

Se presenta el primer informe de un paciente colombiano con una mutación en *MAGT1* asociada con la enfermedad XMEN, con diagnóstico previo de inmunodeficiencia común variable. Se recomienda asesoramiento genético y seguimiento para las familias con casos similares, para permitir la pronta detección de nuevos casos.

Common variable immunodeficiency is considered an inborn error of immunity and, according to the most recent classification of these defects, is categorized as a predominantly antibody deficiency ^1^. Due to the lack of knowledge of the underlying genetic defect in most of the patients ^2^, common variable immunodeficiency disease continues to be a diagnosis of exclusion in immunodeficient patients with increased susceptibility to infections, hypogammaglobulinemia, deficient response to vaccination, or low percentages of switched memory B cells, with no specified genetic defect ^1^^,^^2^. As soon as the genetic defect in these patients is characterized, the subject is re-classified, and efforts could be directed toward a more accurate molecular diagnosis, targeted treatment, and appropriate care ^2^. In the last decade, whole exome sequencing has facilitated the elucidation of the underlying genetic defect in patients with a common variable immunodeficiency disease phenotype ^1^.

Here, we describe the clinical case of a patient with an initial diagnosis of common variable immunodeficiency having a pathogenic variant associated with XMEN disease, discovered for the first time in Colombia after performing whole exome sequencing.

## Case presentation

### Patient and parents

A 26-year-old male and his parents were included in the study. Clinical information and laboratory analysis were obtained from the records of the inborn errors of immunity diagnostic service.

### NKG2D expression

We collected 100 pl of peripheral blood in ethylenediaminetetraacetic acid- anticoagulated Vacutainer^®^ tubes (Becton Dickinson, BD, Franklin Lakes, NJ). Collected blood was incubated with monoclonal antibodies for 20 minutes at room temperature, in the dark, following the manufacturer’s instructions.

The following monoclonal antibodies were used for the staining: anti-CD3- APC (clone UCHT1), anti-CD19-APC (clone HIb 19), anti-CD14-PerCP (clone MφP-9), anti-CD16-APC-Cy7 (clone 3G8), anti-CD56-PE-Cy7 (clone B159), and anti-NKG2D-PE (Clone 1D11). All the antibodies were purchased from Becton Dickinson Biosciences.

After incubating for 20 minutes at room temperature, red blood cells were lysed using 1 ml of FACS^TM^ lysing solution 1X (Becton Dickinson) for 10 minutes at room temperature, followed by two washes with Dulbecco’s phosphate buffered saline 1X (GIBCO - Thermo Fisher Scientific, Waltham, MA).

Cell acquisition was performed with an LSRFortessa^TM^ cytometer (Becton Dickinson), and data was analyzed with the software FlowJo™, version 9.9.6 (https://www.flowjo.com/solutions/flowjo/downloads/v9). Immunophenotyping of natural killer cell subpopulations (CD*3* ^-^/CD16^+^/CD*5*6^+^) was performed as described elsewhere ^3^, and the surface expression of NKG2D was evaluated.

### DNA isolation and whole exome sequencing analysis

Genomic DNA was isolated from blood samples obtained from the patient and his parents using a DNA isolation kit (Puregene^TM^, Gentra Systems, Minneapolis, MN) following the manufacturer’s instructions.

DNA samples were subjected to whole genome sequencing using the Illumina HiSeq™ 2000 platform (Illumina, San Diego, CA) at the Human Genome Sequencing Center of the Baylor College of Medicine in the Texas Children’s Hospital, Houston, TX.

A sequencing yield of 9.1 Gb was obtained, with 91% of the targeted exome bases covered to a depth of at least 20X. Mapping and alignment were performed using the Mercury pipeline of the sequencing center using the human genome reference assembly GRCh*3*7.

Annotated high-quality variants were subsequently filtered to exclude common variants (>1% minor allele frequency) observed in population databases such as the National Heart, Lung, and Blood Institute (NHLBI) Exome Sequencing Project, 1000 Genomes Project, Database of Single Nucleotide Polymorphisms 1*3*5 (dbSNP1*3*5), Exome Aggregation Consortium (ExAC), and internal databases of in-house sequenced exomes. Synonymous and intronic variants in intergenic sequences and untranslated regions (UTR) were also excluded. The deleterious effects of the selected variants were analyzed using Sorting Intolerant from Tolerant (SIFT), Polyphen2, Mutation Taster, and Combined Annotation-Dependent Depletion (CADD).

### Sanger sequencing

The entire coding region and exon/intron boundaries of exon 3 of the *MAGT1* gene were amplified by polymerase chain reaction (PCR) from the gDNA, using the following pairs of primers ^10^: forward 5’- CCTGCTATTGCTTGCTTGG-3’ and reverse 5’-GCAATCCCATTTAATGTCTTATAGC-3’. The thermal cycling profile consisted of one initial cycle of denaturation at 94°C for 5 minutes, followed by 30 cycles at 95°C for 45 s, 62°C for 30 s, 72°C for 45 s, and a final extension cycle at 72°C for 2 minutes.

PCR products (314 base pairs) were verified in a 1% agarose gel and ran at 80V for 45 minutes. The PCR products were then sequenced by Macrogen (Korea), and the results were compared against the *MAGT1* reference gene sequence (NM_032121.5) using the BioEdit™ sequence alignment editor, version 7.2.3 (Ibis Biosciences, Carlsbad, CA).

## Clinical history and laboratory findings

**Figure 1.**
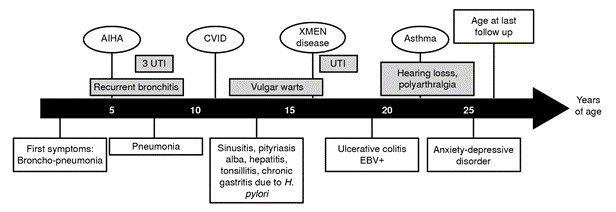
Clinical presentation of the patient with XMEN disease

AIHA: Autoimmune hemolytic anemia; CVID: Common variable immunodeficiency; UTI: Urinary tract infections; EBV: Epstein-Barr virus

We report a male patient, a full-term product of the fourth pregnancy of unrelated healthy parents from San Pedro de los Milagros (Antioquia, Colombia), with complete vaccination records. He is the only male among four siblings. The summarized clinical history of the patient is shown in figure 1. At five months of age, he was hospitalized for four days due to an episode of bronchopneumonia and urinary tract infection, treated with intravenous antibiotics. The bronchopneumonia episodes were recurrent (approximately four episodes per year) until the age of eight.

At the age of five, the patient was hospitalized for 14 days because of a nosocomial urinary tract infection. At this time, a diagnosis of autoimmune hemolytic anemia was also documented. At nine years old, another urinary tract infection was diagnosed and treated. At the age of ten, he presented with community-acquired pneumonia that required 13 days of hospitalization. It is important to point out that two red blood cell transfusions with unknown data and formula were documented, probably due to several episodes of autoimmune hemolytic anemia. 

At 13 years of age, he was hospitalized for an episode of sinusitis, and low serum immunoglobulin levels were reported. An intravenous immunoglobulin replacement therapy was started during this hospitalization. Due to an episode of abdominal pain, hepatopathy was diagnosed, and cholestasis was described in the liver biopsy. Autoimmune hepatopathy was ruled out at the age of 19.

The patient also suffered from skin warts, tinea versicolor (also called pityriasis versicolor) and pytiriasis alba when he was 13 years old. At this time, because of the recurrent respiratory and urinary tract infections, the patient was referred to the *Grupo de Inmunodeficiencias Primarias* in Medellin, Colombia, where they suspected a common variable immunodeficiency, based on the European Society of Immunodeficiencies/ Pan-American Group of Immunodeficiency (ESID/PAGID) criteria ^4^. At the time of diagnosis, the patient exhibited recurrent infections, autoimmune manifestations (hemolytic anemia), and persistent hypogammaglobulinemia (low serum levels of IgG and IgA). T cell subpopulations were reported normal, but B cell subpopulations were abnormal: low percentages of marginal zone-like (IgD^+^/CD27^+^) and switched-memory (IgD^neg^/CD27^+^) B cells were observed at the age of 15 and confirmed at 26 (table 1).

**Table 1:** B lymphocyte subpopulations of the patient by age

B cell subpopulation	At 15 years old		At 25 years old	
	%	Absolute counts (cells/ μl)	%	Absolute counts (cells/ μl)
CD19^+^	37.2^a^	1,575^a^	26.8^a^	492^a^
IgD^+^/CD27^-^	88.0^b^	1,386^b^	52.8^a^	260^b^
IgD^+^/CD27^+^	2.36^b^	37^b^	16.3^a^	80^b^
IgD^-^/CD27^+^	3.88^b^	61^b^	2.2^a^	11^b^
IgD^+^/CD38^+^	2.55	40	0.7	3
IgD^-^/CD38^++^	0.03^b^	1^b^	0.2^a^	1^b^
CD24^++^/CD38^++^	2.53^b^	40^b^	0.7^a^	4^b^

Abnormal values are highlighted in bold.

^a^ Reference values from Schatorje *et al*. ^5^

^b^ Reference values from Morbach *et al*. ^6^

The patient exhibited adequate responses to the *Streptococcus pneumoniae* vaccination (table 2) and rubella protective titers but was not reactive to hepatitis B vaccination. At 17 years old, *Helicobacter pylori* was detected in association with gastric symptoms, and he suffered from urinary tract infections caused by a multi-sensible *Proteus mirabilis*. He was hospitalized when he was 19 years old due to abdominal pain and was diagnosed with ulcerative colitis through a colon biopsy. Epstein-Barr virus (EBV) and *Histoplasma* spp. studies were also performed with the biopsy tissue, finding 28.560 copies/ml (4.46 log) of EBV. Increased plasmatic EBV viral load was also observed (see laboratory findings in table 3). Molecular studies for cytomegalovirus and *Mycobacterium tuberculosis* in the colon biopsy yielded negative results.

**Table 2:** Evaluation of serum IgG vaccination responses to *Streptococcus pneumoniae* in the patient at 13 years of age

Pneumococcal serotype	Pre-vaccination (μg/ml)	Post-vaccination (μg/ml)
1	2.18	4.68
3	**0.74**	**0.73**
44	2.11	4.73
5	8.56	15.28
6B	4.97	8.97
9V	1.78	2.14
14	8.25	11.76
18C	3.38	13.26
19F	10.71	20.49
23F	6.39	9.10

Abnormal values are highlighted in bold. Reference values post-vaccination >1.3 for all ages and serotypes were took from Robbins *et al*. ^7^.

**Table 3:** Additional blood laboratory findings of the patient

Analyte	Min-max values in the clinical record
IgG (1,192 - 2,274 mg/dl)^†^	656 - 1,338*
IgA (107 - 441 mg/dl)^†^	56 - 73
IgM (50 - 439 mg/dl)^†^	105 - 151
ALT (5 - 30 U/L)	40 - 531
AST (0 - 40 U/L)	33 - 237
GGT (9 - 48 U/L)	3 - 27
CPK (0 - 174 U/L)	382
EBV (Log_10_ copies/ml)	4.46 - 5.92

Ig: Immunoglobulin; ALT: Alanine aminotransferase; AST: Aspartate aminotransferase; GGT: Gamma-glutamyl transferase; CPK: Creatine phosphokinase; EBV: Epstein- Barr virus.

Reference values are indicated in parentheses.

* Values at diagnosis under IgG replacement therapy

^†^ Reference values from García de Olarte *et al*. ^8^.

**Figure 2:**
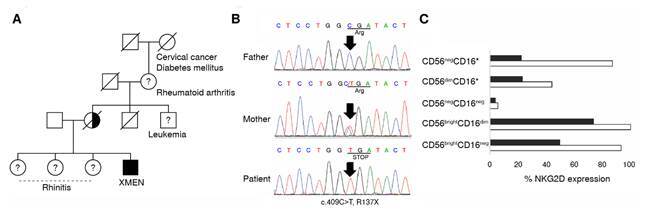
Genetic characterization of the XMEN patient’s family. A) Family pedigree; B) Sanger sequencing of the MAGT1 exon 3; and C) NKG2D expression in the different blood natural killer subsets in the patient (black bars) and a healthy donor (white bars).

The natural killer subsets of the patient showed decreased surface expression of NKG2D (figure 2). When he was 19 years old, he also manifested hearing loss and occasional polyarthralgia. At the age of 25, he reported uncontrolled asthma with an obstructive lung pattern. He was also referred to the psychiatrist with the diagnosis of a mixed anxiety-depressive disorder, exacerbated by his mother’s death due to uterine cancer.

The patient is currently at follow-up every three months but lacks adherence to the *H. pylori* therapeutic scheme and the intravenous immunoglobulin replacement therapy. To date, he has not shown evidence of lymphoma or lymphoproliferative disease.

### Whole genome sequencing and Sanger sequencing analysis

At the age of 18, the patient and his parents were subjected to whole genome sequencing as part of a collaboration to search for monogenetic defects associated with common variable immunodeficiency disease with the Center for Human Immunobiology of the Texas Children’s Hospital, Department of Pediatrics, Baylor College of Medicine, Houston, TX, USA.

After the whole exome sequencing analysis, 31 candidate genes were selected, of which only *MAGT1* was related to the immune system. In the whole exome sequencing filtered data, a hemizygous single base substitution (X:77126382_G>A) introducing a stop codon was identified in the patient. Sequencing the *MAGT1* exon 3 with primers flanking position 29,644 to 29,761 in the *MAGT1* reference gene confirmed the presence of a pathogenic variant in 29,684 (rs387906724, c.409C>T), resulting in a substitution of the arginine in the 137-protein position for a stop codon (R137X). The father has a wild type for this variant, and the mother was a heterozygous carrier. The pedigree of the patient and the inheritance pattern of the variant is shown in figure 2. No consanguinity or maternal miscarriages were documented.

### Clinical phenotype of the patient compared with other reported cases with the same pathogenic variant

We compared the clinical findings of our patient with those reported in five patients with the same mutation ^9^^-^^11^ (table 4). EBV infection has been reported in both of them; associated lymphoproliferative disorders were identified in the other cases but not in the patient reported here. Only our patient presented with pneumonia and bronchitis, urinary tract infections, and gastrointestinal symptoms, such as chronic diarrhea and gastritis. Finally, skin warts were exclusively observed in our patient. These findings confirm the expanded phenotype observed in XMEN patients even if they share the same pathogenic variant in *MAGT1*.

**Table 4:** Clinical phenotype of the XMEN patients with the pathogenic variant c.409C>T

Clinical manifestations	Reported patient (O1)	Li *et al*., (9)* ^B1^	Chaigne-Delalande *et al*. (10)* ^D1^	**Ravell *et al*., (11)** ^*L1^	**Ravell *et al*., (11)** ^*L2^
Age of diagnosis	18	45	16	N/A	N/A
Epstein Barr virus infection	+	+	+	-	-
Lymphoproliferative disorders	-	+	+	+	+
Pneumonia	+	-	-	-	-
Bronchitis	+		-	-	-
Sinusitis	+	-	-	+	+
Streptococcal pharyngitis	-	-	+	-	-
Epiglottitis	-	-	+	-	-
Chronic diarrhea	+	-	-	-	-
Chronic gastritis	+	-	-	-	-
Warts	+	-	-	-	-
Molluscum contagiosum	-	-	-	+	+

N/A: Not available

* B1, D1, L1, and L2 are codes assigned to the XMEN-studied patients by Ravel *et al*. ^11^.

### Ethical considerations

The present study was conducted following the guidelines of the Helsinki Declaration. The family was studied under the protocol 12-07-470 from 2012, approved by the institutional review board at *Sede de Investigaciones Universitarias*, *Universidad de Antioquia* in Medellín, Colombia.

The patient under study, his family, and the healthy control recruited voluntarily accepted to participate, and they all signed the written informed consent. Participant confidentiality and privacy were strictly held in trust by the investigators. Anonymity was maintained when testing the biological specimens.

The patient and his family kept in constant communication with the immunology clinicians involved in the study. The results were explained and communicated, minimizing distress or any potential harm. Reiteration of the participants’ rights to withdraw from the study without penalty was always held. The participants are still in communication with our research laboratory, and we are looking for opportunities to collaborate in search of novel therapies to treat XMEN disease.

## Discussion

We presented the clinical case of the first Colombian patient with XMEN disease, previously diagnosed with common variable immunodeficiency. Over the past 20 years, the diagnostic criteria for common variable immunodeficiency disease have evolved ^1^. At the time of diagnosis, the patient fulfilled the clinical and most paraclinical criteria for this disease ^4^ and exhibited normal responses to pneumococcal vaccination and protective antibodies to rubella. Later, a decrease in switched-memory B cells was documented, an abnormality currently included in the updated common variable immunodeficiency disease diagnostic criteria ^12^.

Common variable immunodeficiency disease is suspected to be a genetic disease in most patients though is unknown whether polygenetic, monogenetic, epigenetic defects, or somatic mutations are responsible for the phenotype ^2^.

The 2022 classification of inborn errors in immunity listed common variable immunodeficiency among the predominantly antibody deficiencies in patients with a severe reduction in at least two serum immunoglobulin isotypes and normal or low number of B cells; an increasing amount of defined genetic defects have been added over time to this category ^1^. However, genetic diseases with a more complex phenotype could also resemble common variable immunodeficiency ^1^.

This is the case of the XMEN, listed among the inborn errors of immunity with susceptibility to EBV and lymphoproliferative conditions together with the autoimmune lymphoproliferative syndrome. Highly heterogeneous clinical manifestations have been reported for XMEN ^11^ and the reported patient was not the exception. This made it difficult to early recognize and diagnose the disease, which is why he was initially classified among a cohort of patients with common variable immunodeficiency disease.

The patient described here was included in an XMEN cohort to further characterize the immunological and clinical phenotype of the disease ^11^. In the mentioned study, several patients, including the one presented here, exhibited high levels of hepatic enzymes, probably non-infectious liver abnormalities, although conventional autoantibodies associated with autoimmune hepatitis have yielded negative results ^11^. Since *MAGT1* has sequence homology with yeast oligosaccharyltransferase, glycoproteomics analysis was performed in the mentioned study ^11^. These studies revealed an N-linked glycosylation defect in multiple immune proteins of XMEN patients. Based on the expanded characterization of the molecular defects underlying the disease, several targeted therapies have been attempted to restore immune function in XMEN patients.

Proper follow-up is fundamental to monitoring EBV-associated neoplasias. Magnesium supplementation does not improve EBV susceptibility and immune dysfunction but restores magnesium intracellular levels in T cells. Recent clinical trials give hope to hematopoietic stem cell transplantation combined with gene editing to treat these patients ^13^^,^^14^. These approaches are possible thanks to the significant advances elucidating the genetic defects underlying the umbrella of inborn error of immunity phenotypes through high- throughput sequencing.

We hope these tools will continue paving the way for further definition of immune-related defects and provide novel approaches to properly care for the patients and their affected families.
